# Nutritional Approaches as a Treatment for Impaired Bone Growth and Quality Following the Consumption of Ultra-Processed Food

**DOI:** 10.3390/ijms23020841

**Published:** 2022-01-13

**Authors:** Shelley Griess-Fishheimer, Janna Zaretsky, Tamara Travinsky-Shmul, Irina Zaretsky, Svetlana Penn, Ron Shahar, Efrat Monsonego-Ornan

**Affiliations:** 1 School of Nutrition Science, Institute of Biochemistry, The Robert H. Smith Faculty of Agriculture, Food and Environment, The Hebrew University, Rehovot 7610001, Israel; shelley.griess@mail.huji.ac.il (S.G.-F.); janna444@gmail.com (J.Z.); tamarmura@gmail.com (T.T.-S.); s94735net@yahoo.com (S.P.); 2 Life Science Core Facilities, The Weizmann Institute of Science, Rehovot 7610001, Israel; ira.zaretsky@weizmann.ac.il; 3 Koret School of Veterinary Medicine, The Robert H. Smith Faculty of Agriculture, Food and Environment, The Hebrew University, Rehovot 7610001, Israel; ron.shahar1@mail.huji.ac.il

**Keywords:** ultra-processed food, growth plate, BMD, supplementation, rescue

## Abstract

The severe impairment of bone development and quality was recently described as a new target for unbalanced ultra-processed food (UPF). Here, we describe nutritional approaches to repair this skeletal impairment in rats: supplementation with micro-nutrients and a rescue approach and switching the UPF to balanced nutrition during the growth period. The positive effect of supplementation with multi-vitamins and minerals on bone growth and quality was followed by the formation of mineral deposits on the rats’ kidneys and modifications in the expression of genes involved in inflammation and vitamin-D metabolism, demonstrating the cost of supplementation. Short and prolonged rescue improved trabecular parameters but incompletely improved the cortical parameters and the mechanical performance of the femur. Cortical porosity and cartilaginous lesions in the growth-plate were still detected one week after rescue and were reduced to normal levels 3 weeks after rescue. These findings highlight bone as a target for the effect of UPF and emphasize the importance of a balanced diet, especially during growth.

## 1. Introduction

In recent decades, there has been an increase in the prevalence of overweight, obesity, and other diet-related chronic non-communicable diseases, such as diabetes, cardiovascular diseases, and non-alcoholic fatty liver [1,2]. Concomitantly with these trends, diets in both high-income and lower-income countries have shifted towards a dramatic increase in the consumption of ultra-processed foods (UPF). These industrially processed products currently make up 75% of world food sales [3].

Food processing is defined as ‘all methods and techniques used by industry to turn whole fresh foods into food products’; this term was first defined by Monterio et al. [4]. Following this definition, a new classification of foods based on the nature, extent, and purpose of their processing, called NOVA, classifies all foods and food products into four groups: unprocessed or minimally processed foods, processed culinary ingredients, processed, and UPF products [5].

UPF products are industrial formulations that typically include substances that are not commonly used in culinary preparations and additives whose purpose is to imitate the sensory qualities of unprocessed foods. Soft drinks, snacks, chips, candies, sweetened milk products, sweetened cereals, and dressings are only a few examples of these products [6]. These products are characterized as having high energy density; greater amounts of free sugar, sodium, fat and saturated fat; and lower amounts of fibers, protein and essential vitamins and minerals, compared to unprocessed or minimally processed foods [2,7].

The consumption of UPF products is a growing phenomenon around the world, with high prevalence in both adults and children. In Canada, up to 61.7% of dietary energy is derived from ultra-processed products [8]. In the UK and the USA, this percentages reach up to 53% [9] and 57.9% [10], respectively. Regarding children, it has been reported that half of US children consume fast food [11]. Moreover, the dietary habits acquired at a young age tend to persist not only into childhood but also into adulthood [12,13]. Beyond the metabolic outcomes of this eating pattern, children are in a critical stage of growth and bone formation. Attaining proper nutrition in the early years of life is crucial to infant growth and development [14,15].

Classic malnutrition—the undernutrition due to scarcity of food or malabsorption—was broadly recognized as being the major cause of longitudinal growth retardation and short stature (stunting) [16]. However, UPF intake is likely to lead to malnutrition due to the low nutritional value combined with the excess of calories, resulting in an inadequate ability to utilize these calories efficiently. This type of malnutrition, which is characterized by multiple nutrient deficiencies, is considered to be a cause of failure to thrive and growth attenuation [17]. However, the effect of excessive UPF consumption on bone development and growth has not been thoroughly studied.

Endochondral ossification is initiated during fetal life and continues postnatally in the growth plates until growth ceases at the period of adolescence [18,19]. This crucial process, which is responsible for bone elongation and is thereby responsible for the longitudinal growth of the whole body, is tightly regulated by genetic and environmental elements. To maximize growth and peak bone mass (PBM), modifiable environmental factors, a balanced diet in particular, should be optimized before the onset of puberty and should be maintained throughout this period of rapid growth. Calcium and phosphorus consist of about 80–90% of bone mineral content [20,21]. Therefore, it is essential to enable a proper intake of those nutrients and others, such as protein, magnesium, zinc, copper, iron, fluoride, vitamins D, A, C, and K for normal bone structure and quality [22].

Recently, Zaretsky et al., established a UPF model in which young rats fed a UPF diet that was rich in fat and sugar suffered from growth retardation due to lesions in their tibial growth plates. Additionally, bone mineral density decreased significantly, and the structural parameters of the cortices and trabecular areas were poor and deteriorated, resulting in the inferior mechanical performance of the entire bone with high fracture risk [23]. Hence, we aim to examine two different approaches in dealing with the growth retardation resulting from UPF-related malnutrition. The first is prevention by supplementing UPF with the micro-nutrients that are vital for the skeleton; the second is rescue approach switches the UPF with balanced nutrition. We hypothesize that the supplementation of UPF or nutritional rescue may prevent the harmful effect of malnutrition caused by an unhealthy and unbalanced diet, which, in turn, impairs proper bone development. The resulting outcomes could shed light upon the effects of malnutrition on bone quality and growth and could determine whether nutritional interventions could help mitigate the effects of UPF consumption.

## 2. Results

### 2.1. Analysis of the Macro and Micro-Nutrient Content of the Control and UPF

**Figure 1:**
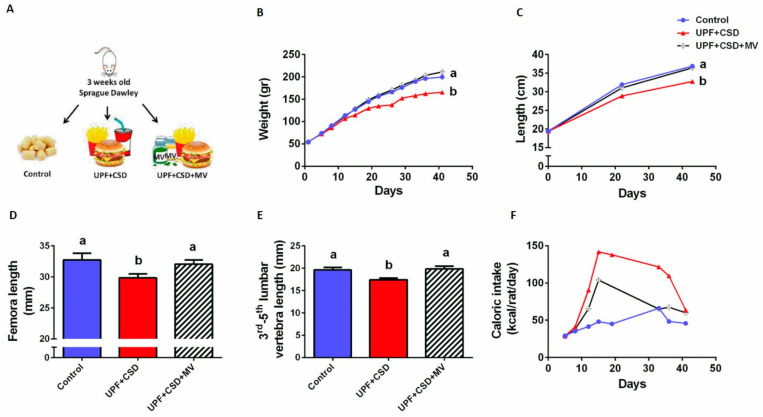
Consumption of ultra-processed food (UPF) leads to growth retardation, which is prevented by supplementation with multi-vitamins and minerals. (**A**) Schematic illustration of the experimental setup. The Control group (n = 8) received a standard diet based on the Harlan Laboratories recommended composition for growing rats; the UPF + CSD group (n = 8) received a diet rich in fat and sucrose, which was composed of a homogenized McDonald’s meal and a soft drink; the UPF + CSD + MV group (n = 8) received a diet rich in fat and sucrose, which was composed of a homogenized McDonald’s meal and a soft drink and was supplemented with multi-vitamins and minerals (n = 8). Throughout the experiments, body weight, body length from the tip of the nose to the end of the tail, and food and fluid consumption were measured twice a week, and daily food intake in kilocalories was calculated for each rat per day. At 9 weeks of age, the rats were anesthetized with isoflurane, blood samples were collected, and they were sacrificed. Their internal organs and bones (femur, tibia, and spine) were harvested. (**B**) Body weight. (**C**) Total length from nose to tail. (**D**) Femora length at 9 weeks of age. (**E**) Third to fifth lumbar vertebra length at 9 weeks of age. (**F**) Daily caloric intake (Kcal/rat/day). Values are expressed as mean ± SD, n = 8. Different letters denote significant difference at *p* < 0.05 between groups.

An analysis of the macro and micro-nutrient content of the Control and UPF diet showed similar levels of protein, a higher fat content in the UPF (Supplementary Figure S1A), and major differences between the micronutrient composition of the two diets: Ca, phosphorus, zinc, iron, magnesium, and Cu were significantly lower in the UPF (Supplementary Figure S1B). Thus, we supplemented the UPF with micro-nutrients, creating three experimental groups: Control, UPF with caloric soft drink (UPF + CSD group), and UPF + CSD supplemented with multi-vitamins and minerals (UPF + CSD + MV group) (Figure 1A).

### 2.2. The Effect of Micro-Nutrient Composition on Growth and Physiological Parameters

During the experiment, the rats’ body weight (BW) and total length from the tip of the nose to the end of the tail were measured. The femora and vertebrae were measured using a µCT device following the sacrifice of the rats. The BW, total length, femora, and vertebrae length demonstrated the harmful effect of the UPF. The rats suffered from severe stunting phenotype starting as early as after 3 weeks of feeding, which presented as retardation in normal growth and development (Figure 1B–E). However, the supplemented group, UPF + CSD + MV, was equal in those parameters compared to the Control group and presented a normal growth pattern compared to the rats who solely received UPF (Figure 1B–E).

During the experiment, food and fluid consumption were measured, with both the UPF-based groups showing higher caloric intake compared to the Control group, with the highest caloric intake being observed in the UPF + CSD group (Figure 1F).

A calculation of Ca, zinc, iron, magnesium, and Cu intake was performed for each experimental diet compared to the Control. The higher intake of food in the UPF + CSD group managed to increase the intake of zinc, magnesium, and iron, despite their low content in the diet. However, the higher consumption did not compensate for calcium and copper deficiencies (Supplementary Figure S2A–E).

#### 2.2.1. The Effect of UPF and Supplementations on the Metabolic Profile of the Rats

To assess the dietary effects on the rats’ metabolic state, blood samples were analyzed for biochemical and endocrine profiles. The most dramatic effect was observed in the UPF + CSD group, particularly on the bone-related parameters (Supplementary Table S1). Phosphate and ALP were higher, and calcium levels were lower from the normal range and differed significantly from the Control. These parameters were corrected upon supplementation. Moreover, the levels of creatinine phosphokinase and aspartate transaminase, which are indicative of damage to the muscle, were above their normal range and differed significantly from the Control. Once again, their values were normalized upon supplementation (Supplementary Table S1).

Serum levels of the metabolic hormones ACTH and leptin did not differ between the groups. Nonetheless, the levels of the parathyroid hormone (PTH), the plasma calcium balancing hormone, were significantly elevated in the UPF + CSD rats compared to in the Control group, probably due to the low levels of serum calcium. Consistently, the profile of the bone related hormones reflected the damaged bone condition. Serum levels of sclerostin, DKK1, and Fibroblast Growth Factor 23 (FGF23), all of which are produced by osteocytes and serve as markers for bone metabolic status [24,25], were significantly reduced in the UPF-consuming rats compared to the Control group and were restored by the addition of micro-nutrients (Supplementary Table S1). Taken together, we have shown that supplementation with MV was able to prevent many of the harmful effects of UPF on bone.

#### 2.2.2. The Effect of UPF and Supplementations on the Rats’ Bone Properties

**Figure 2:**
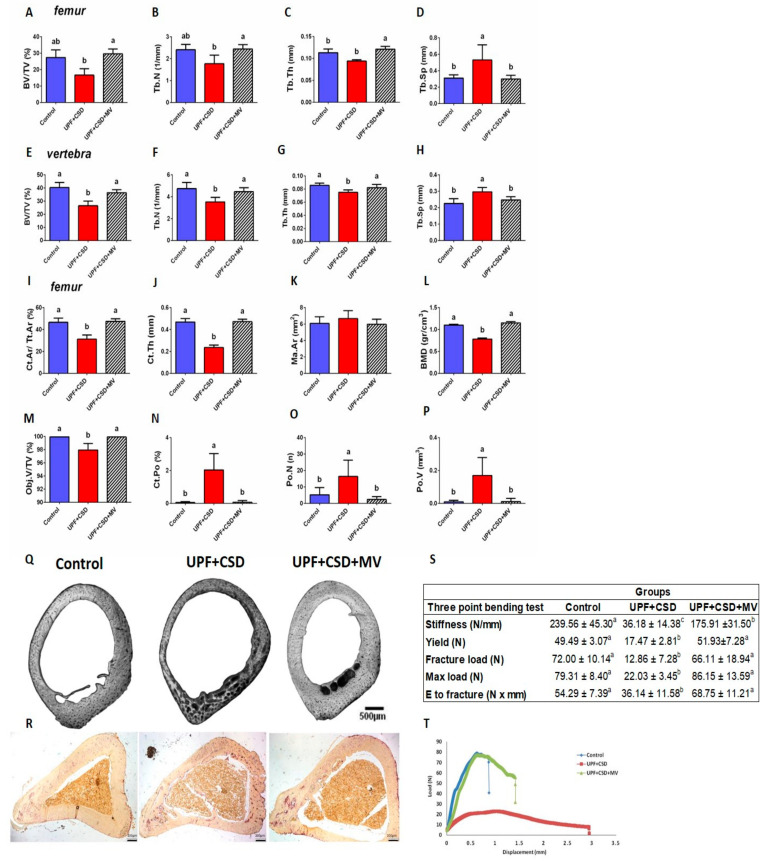
Supplementation of ultra-processed food (UPF) with multi-vitamins and minerals restores bone morphometry and mechanical properties. Control and UPF groups were compared to a group receiving an ultra-processed food (UPF) diet with multi-vitamin and minerals (UPF + CSD + MV). Femora bones were scanned in a SkyScan 1174 X-ray computed microtomograph scanning device and were analysed for trabecular and cortical properties. (**A**–**H**) Trabecular parameters: bone volume fraction (BV/TV), trabecular thickness (Tb.Th), trabecular number (Tb.N), and trabecular separation (Tb.Sp). (**I**–**L**) Cortical bone parameters: cortical area fraction (Ct.Ar/Tt.Ar), average cortical thickness (Ct.Th), medullary area (Ma.Ar), and bone mineral density (BMD). (**M**–**P**) Bone porosity parameters: percent object volume (Obj.V/TV %), cortical porosity (Ct.Po%), pore number (Po.N), and total pore volume (Po.V mm^3^). (**Q**) Light microscopy images of cross-sections of rat cortical bone representing cortical bone porosity. (**R**) TRAP staining of cross sections of the cortical bone of the tibia. (**S**) Biomechanical parameters assessed by three-point bending test: stiffness (N/mm), yield (N), fracture load (N), max load (N), and energy to fracture (N × mm). (**T**) Representative load–displacement curves. Values are expressed as mean ± SD, n = 8. Different letters denote significant difference at *p* < 0.05 between groups.

In order to estimate the bone trabecular and cortical properties, the femora and the 5th lumbar vertebrae bones were scanned using a µCT device. Trabecular bone analysis of the femora demonstrated that the UPF + CSD group had lower values, though not significant, in terms of the bone volume fraction (BV/TV%), trabecular thickness (Tb.Th), and trabecular number (Tb.N) compared to the Control group (Figure 2A–C). Trabecular separation (Tb.Sp), which represents the mean distance between trabeculae, was significantly higher in the UPF + CSD group vs. the Control (Figure 2D). Upon supplementation, bone properties were similar to the Control and were significantly improved compared to UPF + CSD in all trabecular parameters. To understand the effect of UPF and supplementation on additional bones, the trabecular parameters of the 5th lumbar vertebrae were analyzed. The trabecular parameters of the vertebra were comparable to the femur. BV/TV, Tb.N, and Tb.Th were reduced in the UPF + CSD and were higher in the Control and UPF + CSD + MV groups, while Tb.Sp was higher in the UPF + CSD group (Figure 2E–H).

Cortical bone analysis showed that the UPF + CSD group had a significant decrease in the ratio between the cortical and total area Ct.Ar/Tt.Ar. Cortical thickness (Ct.Th) was only 50% compared to the Control. Supplementation resulted in equal levels of cortical bone parameters, including bone mineral density (BMD), which resembled that of the Control group (Figure 2I–L). The most dramatic finding was the remarkable porosity seen in the cortices of the bones belonging to the rats consuming UPF + CSD. Even a cursory look at the typical cross sections of the bones immediately revealed the sieve-like appearance of the cortices of the UPF + CSD group compared to the normal looking cortices of the other two groups. Quantitative analysis showed that the number of pores (Po.N) was 3 times higher in the UPF + CSD than it was the Control group. Similarly, the total volume of pores (Po.V) increased by 17-folds (Figure 2M–Q). TRAP staining of the cortical bone fraction revealed increased osteoclast number upon UPF consumption (Figure 2R), presumably to maintain normal serum calcium levels. This osteoclast over-activation may account for the sieve-like appearance demonstrated in the cortex of rats fed with the UPF diet.

The three structural parameters with the most significant effect on the mechanical performance of bones, are the geometry, mineral density, and porosity of the cortical bone. The results presented above led us to expect that supplementation could improve the mechanical strength of the femur. Indeed, a three-point bending test of the femora bones showed that unlike the bones from the UPF + CSD group, which demonstrated poor mechanical properties, supplementation resulted in bones with high mechanical performance that was as good as the Control group, other than a partial correction of the bone stiffness (Figure 2S,T).

**Figure 3:**
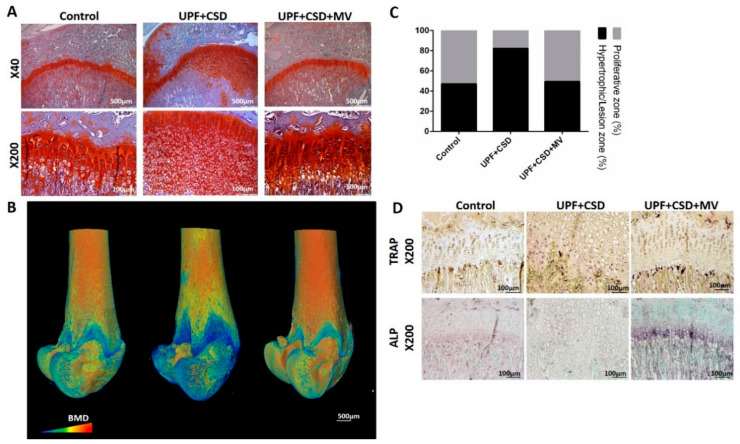
Consumption of ultra-processed diet results in damaged growth plate (GP) and modified chondrocyte-differentiation process. Tibiae from Control, UPF + CSD, and UPF + CSD + MV groups were dissected, processed, embedded in paraffin blocks, and stained with (**A**) safranin-O staining. (**B**) Representative 3D images of femur bones visualized by Amira software. (**C**) Quantification of the relative ratio of the proliferative and hypertrophic zones in the GP. (**D**) TRAP and ALP staining of tibial GP from Control, UPF + CSD, and UPF + CSD groups.

Abnormal bone phenotypes may stem from interference with the endochondral ossification process taking place in the GP [26]. Sections stained with Safranin-O (which distinguishes cartilage from bone), revealed a significant and uneven widening of the GP in rats belonging to the UPF + CSD group. This widening was characterized by a mass of avascular non-mineralized cartilage lesions that extended from the epiphyseal GP into the metaphysis of the tibia [23]. The normal GP phenotype was observed in the Control group and in the group with the supplemented diet, in line with the normal bone phenotype (Figure 3A). Three-dimensional µCT reconstructions of the entire femur with a color scale indicating mineral density also exhibited a greater non-mineralized cartilage lesion areas in the GPs of the UPF + CSD group, which was corrected upon supplementation (Figure 3B). Quantification of the width of the different zones of the GP showed the enlargement of the non-proliferative cartilage as a result of the consumption of a UPF diet, while in the supplemented group, the proportions became normal (Figure 3C). Additional stainings were performed to characterize the cells within the lesion. The activity of the ALP enzyme, which is expressed by hypertrophic chondrocytes and is involved in the initiation of matrix calcification, demonstrated negligible staining in the GP of bones from the UPF + CSD group compared to the other groups, suggesting the inhibition of terminal differentiation between chondrocytes (Figure 3D). Osteoclastic activity studied by TRAP staining only revealed limited activity in the UPF + CSD group in the lesion area, and more activity in the junction between the cartilaginous lesion and the ossified bone. The Control group demonstrated a continuous presence of osteoclasts in the chondro–oseous junction, where cartilage is replaced by bone tissue. TRAP activity in the supplemented group was once again similar to the Control (Figure 3D).

#### 2.2.3. The Effect of Supplementations with Calcium or Copper on Growth and Metabolic Status

**Figure 4:**
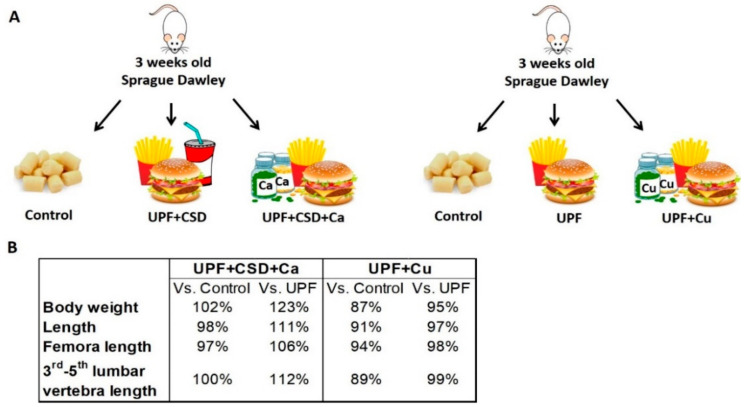
Supplementation of ultra-processed diet with calcium (Ca) prevents damage to growth, whereas supplementation with copper (Cu) does not prevent damage. (**A**) Schematic illustration of the experimental setup. (**B**) The physiological parameters (body weight, length, femora and third to fifth lumbar vertebra length) of the supplemented groups compared with Control or the UPF based groups.

The UPF + CSD group consumed less calcium and copper throughout the experiment and did not manage to compensate for the deficiencies in the other minerals (Supplementary Figure S2). Hence, we tested whether the supplementation of the UPF with Ca or Cu alone could prevent skeletal damage (Figure 4A). Supplementation with Ca resulted in a normal growth pattern in terms of body weight, total length, and femora and 3rd–5th lumbar vertebra length (Figure 4B). However, the rats fed with Cu supplementation were lighter and shorter than the Control and even slightly lighter and shorter than they were in the UPF group. The final length of the rats in the UPF + Cu group was 91% of the length of the Control groups and 97% of the length of the UPF group. The femora and the lumbar vertebra lengths exhibited the same pattern, in which the length of those bones was the shortest (94% and 89% of the length of the Control group, respectively).

#### 2.2.4. The Effect of Ca or Cu Supplementations on Rat Bone Properties

The UPF + CSD group had lower trabecular bone parameters compared to the Control group. Supplementation with Ca led to higher levels but without significant BV/TV% and Tb.N from the Control or UPF + CSD group. The major improvement upon supplementation was in Tb.Th, where the UPF + CSD + Ca group differed significantly from both the UPF + CSD and the Control groups. As a result, Tb.Sp was lower in the Control group and in the Ca-supplemented groups than it was in the UPF + CSD group (Table 1). Analysis of the trabecular bone of the fifth lumbar vertebra revealed the same trend of effects with some dissimilarities. UPF + CSD + Ca managed to reach Control levels in terms of Tb.Th. However, BV/TV%, Tb.N and Tb.Sp were only partially rescued and did not reach the Control levels (Table 1).

Regarding cortical bone parameters, a diet supplemented with Ca corrected the Ct.Ar/Tt.Ar and the average Ct.Th. Interestingly, Tt.Ar was smaller and similar to the UPF + CSD group, but the medullary area (Ma.Ar) was significantly lower in this group, resulting in a higher ratio between Ct.Ar to Tt.Ar. Additionally, Ca Supplementation fully rescued and even improved the mineral density of the cortical bone and its porosity (Table 1). The improvement in the cortical structure resulted in full improvements in the bone mechanical performance in a three-point bending test (Table 1).

**Figure 5:**
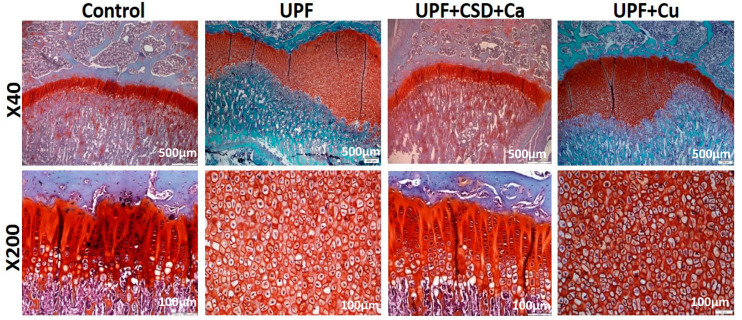
Consumption of ultra-processed diet results in a damaged growth plate (GP), and supplementation with calcium restores it while copper does not. Tibiae from the Control, UPF, UPF + CSD + Ca, and UPF + Cu groups were dissected, processed, embedded in paraffin blocks, and stained with safranin-O.

Concomitantly with the improvement in the bone structure, the serum levels of key bone state indicators were normalized. Supplementation managed to reduce the levels of phosphate and ALP and to elevate Ca to Control levels and repair the bone endocrine profile (PTH, DKK1, sclerostin, and FGF23) (Supplementary Table S2). Safranin-O staining demonstrated a normal GP phenotype and width, as seen in the Control group (Figure 5).

Supplementation with Cu did not improve the bone trabecular parameters at all, and they were similar to those of the UPF group (Table 1).

Ct.Ar/Tt.Ar, Ct.Th, and Ma.ar resembled the UPF group. The BMD was even lower than that of the UPF group and was 34% lower than that of the Control group. The only parameter that slightly improved was cortical porosity (3.30 ± 1.15 compared to 4.61 ± 1.29) although it was still higher than that of the Control, but Po.N and Po.V did not differ from the UPF group. The three-point bending test of the femoral bones exhibited the deteriorated state of the bones once again, demonstrate lower values of mechanical performance, similar to the UPF group (Table 1). An analysis of the blood levels of Ca, P, and ALP reflected in the bone state, with low levels of Ca in the serum and elevated levels in the ALP and phosphorus. Moreover, the levels of the liver enzymes ALT and AST were elevated upon Cu supplementation, indicating damage to the liver (Supplementary Table S2). Histological analysis of the GP was compatible with the deteriorated growth and bone patterns, as the GP of the Cu-supplemented rats had lesions within the GP with s enlarged width, similar to the UPF group (Figure 5).

Taken together, these results demonstrate that supplementation with either a multi-vitamin and minerals or even with Ca alone was able to rescue most of the deteriorated bone phenotypes generated by the consumption of the UPF diet.

#### 2.2.5. The Effect of the Different Diets and Supplementations on the Kidneys State

**Figure 6:**
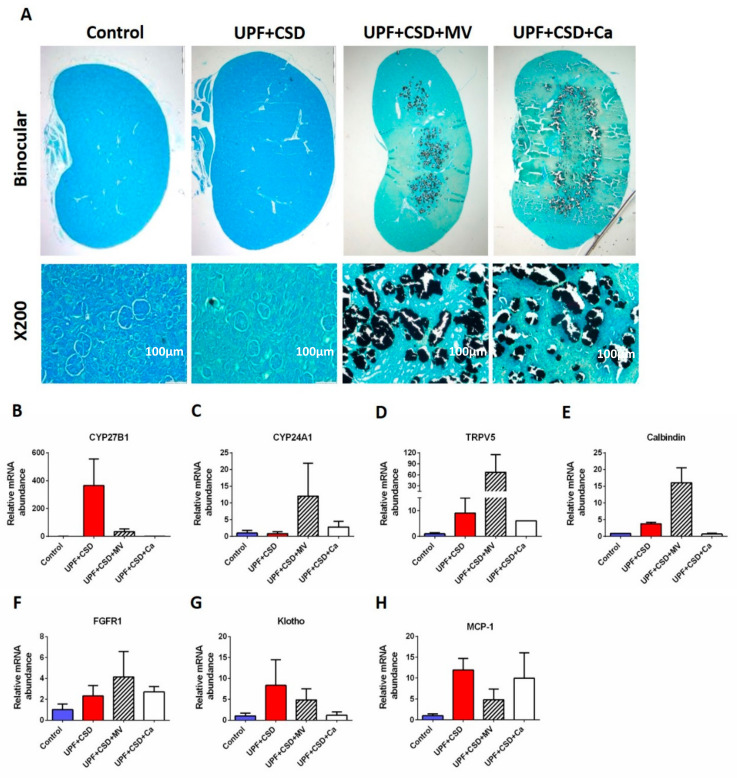
Supplementation of ultra-processed diet results in formation of mineral deposits in the kidneys. (**A**) Kidneys from Control, UPF + CSD, UPF + CSD + MV, and UPF + CSD + Ca groups were dissected, processed, embedded in paraffin blocks, and stained with Alcian Von Cossa staining. (**B**–**H**) An amount of 100 mg of kidney tissue was collected and homogenized in TRI Reagent for RNA extraction. Following the conversion of mRNA to cDNA, the expression level of mRNA was quantified with real-time PCR for the selected genes involved in the control and metabolism of vitamin D.

Histological analysis of the rats’ kidneys revealed a formation of mineral deposits in the groups, which received supplementation with multi-vitamins and minerals or Ca (Figure 6A). To further elucidate this occurrence and its implication, we focused on Ca and vitamin D3 metabolism in the kidney. Serum levels of 1,25-(OH)_2_D_3_ are tightly regulated by feedback loops through the renal expression of CYP27B1, an activator of vitamin D, and CYP24A1, which degrade it [27,28]. Concomitantly, in the UPF + CSD group, the up regulation in the CYP27B1 mRNA (by 252.89-folds) and the down regulation of the CYP24A1 mRNA (by 0.82-folds) compared to the Control group were observed (Figure 6B,C). Moreover, in response to the hypocalcemia in the UPF + CSD group, PTH levels were elevated to 2584.02 pg/mL compared to a level of 581.2 pg/mL in the Control group (Supplementary Table S2). Following the modifications in the vitamin D-related genes and presumably its activation, an up regulation in the expression levels of the vitamin D downstream genes, TRPV5 and calbindin in the kidney, that are connected to Ca reabsorption was detected (Figure 6D,E).

FGF23, the regulator of phosphate homeostasis, is produced by osteocytes and target the kidney to reduce 1,25-(OH)_2_D_3_ levels by inhibiting renal CYP27B1 expression and by promoting CYP24A1 expression38. Serum levels of FGF23 were decreased in the UPF + CSD compared to in the Control group (480.3 pg/mL vs. 1812.5 pg/mL) due to the reduced bone tissue. FGF23 acts via a Klotho:FGF receptor-1 complex in the kidney and creates negative feedback regulation by inhibiting the expression of its own receptor. Indeed, the lower serum levels of FGF23 in the UPF + CSD led to increased expression levels of Klotho and FGFR1 by 8.32- and 2.32-folds, respectively (Figure 6F,G).

The UPF + CSD + MV group showed a different gene expression pattern compared to the UPF + CSD group, probably due to the adequate levels of Ca in the diet among other micro-nutrients, including vitamin D (Figure 6B–G). The UPF + CSD + Ca group, which received supplementation with Ca phosphate, had similar hormonal levels of FGF23 (1540.9 pg/mL) and PTH (624.9 pg/mL) to the Control, which were higher than those found in the UPF + CSD + MV group (Supplementary Table S1B). The higher levels of FGF23 in the serum resulted in lower levels of Klotho expression, which was also the case in the Control group (Figure 6G). This group had marginal elevation in the levels of CYP27B1 (by 1.45-folds) and elevation in the mRNA expressions of CYP24A1 (by 2.8-folds). This moderate increase in genes related to the activation of vitamin D may also result in the modest expression of TRPV5 and Calbindin (Figure 6B–E). These similarities in the expression pattern between UPF + CSD + MV and the Control is reflected by the normal levels of serum calcium (11.5 mg/dL).

It is important to highlight the elevation in the expression levels of macrophage chemoattractant factor-1 (MCP-1) in the UPF fed groups compared to in the Control group. This marker, which is indicative of renal epithelial injury, was elevated in the UPF + CSD group (by 11.94-folds). Interestingly, the levels of this marker remained elevated upon supplementation based on the UPF. We can see that upon renal injury, supplementation resulted in the presence of mineral deposits in the kidneys of those groups (Figure 6H).

#### 2.2.6. Rescue Experiment: Short or Prolonged Transition to Balanced Nutrition

**Figure 7:**
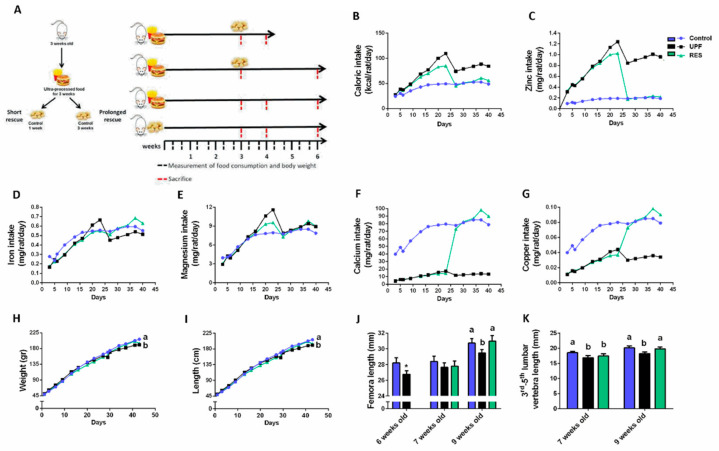
Transition from exclusive ultra-processed food (UPF) consumption to a balanced diet leads to a compatibility between the caloric intake and micro-nutrient consumption and catch-up growth. (**A**) Schematic illustration of the experimental setup. Exclusive consumption of UPF for 3 weeks was followed by consumption of the Control diet before growth cessation in short and prolonged repair during accelerated growth (1 week and 3 weeks of control diet following 3 weeks of UPF diet). Throughout the experiments, body weight, body length from the tip of the nose to the end of the tail, and food consumption were measured twice a week, and daily food intake in kilocalories was calculated for each rat per day. At 6, 7, and 9 weeks of age, the rats were anesthetized with isoflurane, blood samples were collected, and they were sacrificed. Their internal organs and bones (femur, tibia, and spine) were harvested. (**B**) Daily caloric intake (Kcal/rat per day). (**C**) Daily zinc intake (mg/rat per day). (**D**) Daily iron intake (mg/rat per day). (**E**) Daily magnesium intake (mg/rat per day). (**F**) Daily calcium intake (mg/rat per day). (**G**) Daily copper intake (mg/rat per day). (**H**) Body weight. (**I**) Total length from nose to tail. (**J**) Femora length at 6, 7, and 9 weeks of age. (**K**) Third to fifth lumbar vertebra length at 7 and 9 weeks of age. Values are expressed as mean ± SD, n = 8. * Significantly differ from control at same age. Different letters denote significant difference at *p* < 0.05 between groups.

After investigating the ability of supplementation with micro-nutrients to prevent the harmful effect of the UPF diet on bone development, we aimed to study whether a transition to a balanced diet could rescue already existing damage. To this end, we performed an in vivo experiment in which UPF was consumed exclusively for 3 weeks followed by the consumption of a Control diet before growth cessation. We examined short (1 week—RES1W group) and prolonged (3 weeks—RES3W group) rescue during the accelerated growth period (Figure 7A).

The caloric intake of the UPF group was once again higher than that of the Control group, and the replacement of the UPF by the Control diet resulted in the reduction of the caloric intake associated with the replenishment of mineral needs (Figure 7B–G). During the short rescue period, the weight did not differ between all groups (Figure 7H). Additionally, one week of a balanced diet did not lead to a catch up in the length, and the RES1W did not differ from that of the UPF group in terms of the total length as well as in the length of the femur and vertebra (Figure 7I–K). However, in the remaining two weeks of the experiment, the UPF continued to demonstrate pronounced growth retardation, while the prolonged rescue managed to correct body weight and length, femora, and vertebra length, to the levels of the Control group (Figure 7H–K).

**Figure 8:**
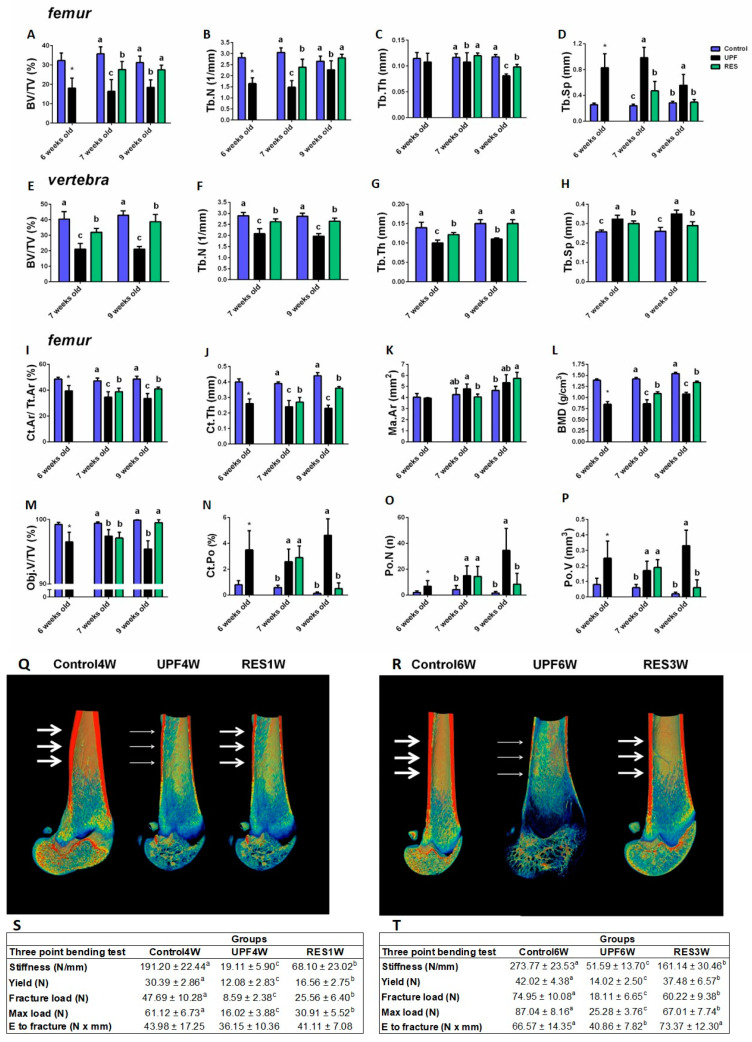
Short or prolonged transition to a balanced diet does not lead to a full recovery of cortical and trabcular bone or the bio-mechanical strength. Control and ultra-processed food (UPF) groups were compared to a group that received a Control diet for 1 week and 3 weeks following 3 weeks of a UPF diet (RES). (**A**–**H**) Trabecular parameters: bone volume fraction (BV/TV), trabecular number (Tb.N), trabecular thickness (Tb.Th), and trabecular separation (Tb.Sp). (**I**–**L**) Cortical parameters: cortical area fraction (Ct.Ar/Tt.Ar), average cortical thickness (Ct.Th), medullary area (Ma.Ar), and bone mineral density (BMD). (**M**–**P**) Bone porosity parameters: percent object volume (Obj.V/TV %), cortical porosity (Ct.Po%), pore number (Po.N) and total pore volume (Po.V mm^3^). (**Q**,**R**) Representative 3D images of femur bone cross-sections visualized by Amira software, with arrows indicating cortical thickness. (**S**,**T**) Biomechanical properties: stiffness (N/mm), yield (N), fracture load (N), max load (N), and energy to fracture (N × mm) assessed by three-point bending test. Values are expressed as mean ± SD, n = 8. * Significantly differ from control at same age. Different letters denote significant difference at *p* < 0.05 between groups.

Analysis of the trabecular bone of the femur revealed that short rescue enabled full Tb.Th correction. However, BV/TV%, Tb.N, and Tb.Sp were only partially improved. In the prolonged rescue, all trabecular bone parameters except Tb.Th reached the Control levels. The difference in the Tb.Th between short and prolonged rescue may be indicative of the pattern of response of the trabecular bone to nutritional transition (Figure 8A–D). Trabecular analysis of the fifth lumbar vertebra showed a different pattern than the femur. Both short and prolonged rescue led to partial improvement in the trabecular parameters (Figure 8E–H).

All cortical parameters, Ct.Ar/Tt.Ar, Ct.Th, and BMD, were only partially corrected, and the same pattern was seen for short and prolonged rescue, implying that trabecular and cortical bone had different responses (Figure 8I–L). The reduced Ct.Th in the UPF and rescue groups is visualized with representative 3D images of femur bone cross-sections, with arrows indicating cortical thickness (Figure 8Q,R).

Although prolonged rescue did not fully improve cortical parameters, porosity levels of the cortical bone were reduced and were used as a Control, while in the short rescue, they were the same as they were in the UPF group (Figure 8M–P, Supplementary Figure S3). The mechanical performance of the femoral bones had already been improved after a week of balanced nutrition but still did not reach the levels of the Control group. This pattern was sustained during the longer rescue period as well (Figure 8S,T, Supplementary Figure S4).

Blood analysis of the RES1W revealed an elevation in the Ca levels in the blood and a reduction in the phosphorus and ALP levels. The Ca and ALP levels were the same as those in the Control group, and phosphorus levels did not differ from those found in the Control and UPF groups. In the prolonged rescue period, all of these parameters reached Control group levels (Supplementary Table S2).

**Figure 9:**
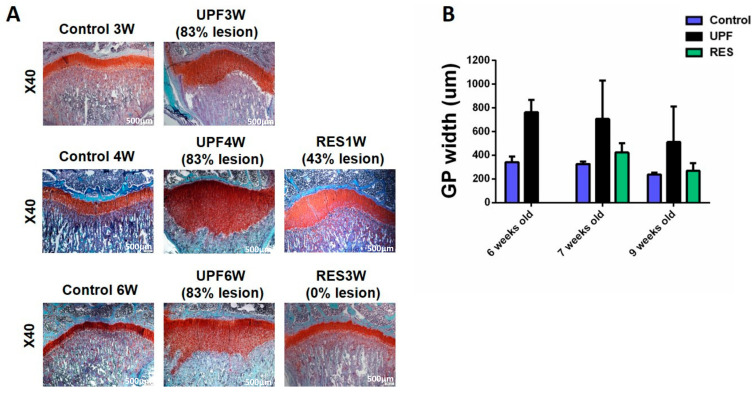
Short or prolonged transition to a balanced nutrition recovers the damage to the GP following UPF. (**A**) Tibiae from Control, UPF, and RES groups were dissected, processed, embedded in paraffin blocks, and stained with safranin-O. (**B**) The width of the whole GP was measured at ten different points along the GP, averaged for each GP, and then averaged with measurements from other GP samples in each group.

GP analysis revealed a marked cartilaginous plaque and increased width already after three weeks of feeding with UPF. The average width of the UPF3W group (762.68 ± 105.54) was two times more than the Control3W group (342.71 ± 45.38). The major difference in the total GP width resulted from enlarged hypertrophic zone width. Short rescue reduced the width of the GP, and the percentage of the GPs with lesion declined from 83% in the UPF4W group to 43% in the RES1W. Longer rescue resulted in a normal appearance of GP and width as Control without any lesions (Figure 9).

Taken together, while short and prolonged rescue improved growth, in terms of weight, longitudinal growth, and the disappearance of lesions in the GP, bone quality was not completely improved regarding the trabecular and cortical parameters and the mechanical performance of the femur. These results emphasize that not all types of damage are reversible, even by a transition to a balanced nutrition.

## 3. Discussion

To date, it is well established that UPF consumption leads to a variety of deeply rooted metabolic complications, including overweight, obesity, cardiovascular diseases, insulin resistance, diabetes, nonalcoholic fatty liver disease, etc. [15,29]. In a previous project conducted in our lab, young rats that were fed with a diet consisting exclusively of UPF suffered from severe skeletal development damage [23].These concerning results and the elevated rates of UPF consumption among children have prompted us to examine the possibility of nutritional interventions to deal with this issue. Here, we showed the positive effect of supplementation with multi-vitamins-minerals on bone growth and quality that was followed by damage to the rats’ kidneys with modifications in inflammation and vitamin-D metabolism. We further demonstrated that a nutritional rescue approach partially improved the structural and mechanical parameters of bone.

Our first approach was to supplement the UPF diet with multi-vitamins and minerals. Supplementation led to normal growth pattern, GP morphology, and structural bone parameters. However, the mechanical strength of the femur bone revealed the single effect of diet supplemented with multi-vitamins and minerals: a reduction in the stiffness. Stiffness is a measure of the resistance offered by the whole bone to the applied displacement over the elastic segment. The meaning of this result is that concerning the UPF + CSD + MV, less force is required to produce a given displacement [30]. Collagen and mineral are the two composite materials of the bone tissue and contributes to its mechanical behavior differently. Their amount, arrangement, and molecular structure determine the mechanical properties of the bone. The mineral component is directly related to the material strength and stiffness of the tissue, while the collagen phase contributes to the toughness of the tissue and improves the bone’s work to failure [31,32]. These results may imply that supplementation with multi-vitamins and minerals did not fully restore the mineral component of the bone.

During the experiment, the UPF + CSD group reached higher food and caloric intake levels. This is probably due to the high palatability of the diet and the attempt to attain higher amounts of the micronutrients. Following analysis of the micro-nutrient consumption along the experiment, the UPF + CSD had a higher intake of many minerals due to increased food consumption, but not calcium or copper, which are essential components in bone growth and structure. These results led us to a follow-up experiment where UPF was supplemented with calcium or copper alone.

While calcium supplementation led to favorable results for the femoral bones, the trabecular morphology of the vertebra was less favorable. BV/TV% as well as Tb.N and Tb.Sp did not reach the same levels as the Control group. This may imply that supplementation does not affect all bones in the same manner. Bagi et al. claimed that the structural properties of the trabecular bone in the vertebra and femur are different because of the difference in loading [33]. Rats are quadrupeds, meaning that the femur is likely to receive more mechanical loading than the lumbar spine. Several mechanisms relating to bone growth stimulation by loading are described, for instance, the anabolic pathways of the Wnt/β-catenin in osteocytes which transduce the signals of mechanical stimuli to the osteoblasts [34]. Another mechanism occurs is via the periosteal osteoblasts that exhibit greater mechano-sensitivity compared to endosteal osteoblasts [35]. Studies have repeatedly shown that periosteal bone formation following mechanical loading occurs primarily in regions where strains are high and occur less where peak strains are lower [36]. Moreover, periosteal bone formation rates differ among skeleton sites, suggesting that periosteum anatomy or regulation may differ throughout the axial skeleton [35].

A histological analysis of the rats’ kidneys revealed the formation of mineral deposits in the groups who received supplementation with minerals or a vitamin mix or just Ca. Along with this, an elevation in MCP-1 mRNA levels was detected in the kidneys of the rats consuming the UPF diet. A review published by Odermatt et al. showed that a Western-style diet characterized by highly processed and refined foods and high contents of sugars, salt, fat, and protein from red meat led to the impairment of renal vascular function, inflammation and subsequent microalbuminuria, and a rapid decrease in kidney function [37]. Additionally, based on UPF diet, supplementation with MV or Ca resulted in the formation of mineral deposits in the rats’ kidneys. This presence of mineral deposits in the medulla of the kidney triggers renal epithelial cells to produce MCP-1 [38]. The review by Heaney et al. addresses how most studies show no increase in stone risk with high Ca intake (from either diet or supplements) [39]. However, it has been shown that supplementation with Ca can also increase the risks of acute gastrointestinal events, kidney stones, and cardiovascular diseases such as myocardial infarction and stroke [40,41]. Therefore, supplementation is not always the solution for an extremely unbalanced diet. Moreover, it is important to understand that such a diet is not only deficient but that it is also fortified with a variety of un-nutritional factors, and this combination can be harmful.

The low levels of Cu in the UPF diet led us to assume that the supplementation of Cu could have an impact since it has a role in collagen maturation and hence bone composition and structure. Lysyl oxidase is the Cu containing enzyme that catalyzes the crosslinking of the epsilon amino groups of lysine and hydroxyproline between adjacent collagen fibrils, thereby increasing its mechanical strength [42]. Unpredictably, supplementation with copper did not improve neither growth nor bone quality and even led to a lower BMD compared to the UPF group. these results suggest that copper deficiency was not the primary cause of the phenotype.

Another approach to deal with the damage caused by UPF on growth is replacing the UPF with a balanced diet that is suitable for growth for the catch-up growth period. This approach led to a normal growth pattern in terms of weight, total length, and femoral length, resulting in normal growth pattern that were the same as those in the Control group. Growth stunting constitutes the most common effect of malnutrition. When the primary cause of malnutrition is resolved, catch-up growth usually occurs [43]. Moreover, trabecular parameters were significantly greater in the rescue period than they were in the UPF group both in the short- and long-term catch-up periods. However, cortical bone quality and BMD only demonstrated partial catch-up. In research published by Pando et al. [43], SD male rats aged 24 days old were subjected to ten days of 40% food restriction followed by refeeding for a 1 or 26 day catch up. As in our study, not all bone parameters were corrected upon refeeding. Additionally, both short- and long-term rescue had better mechanical strength than that of the UPF groups, but they did not achieve full correction.

Evidence that links UPF eating patterns to skeletal development is still scarce. Nevertheless, a recent study has explored these relationships during childhood. The authors show that BMD at birth and at the age of 4 and 6 years old were lower in those living close to fast-food outlets compared to children residing in proximity to healthy specialty stores. Another study comparing different dietary patterns showed that a diet based on fruits, milk, and whole grains was positively associated with BMD in adults compared to a fast-food pattern eating group [44]. Additionally, evidence from the Framingham Osteoporosis Study suggests that high candy consumption was associated with low BMD in adult men and women [45].

In this study, we provided young growing rats with a whole diet that was equivalent to that eaten by humans. On one hand, it is a novel scientific approach. The common nutritional methodology is to add or remove one component at a time from the diet and to study its effect separately, as in pharmaceutical or genetic studies. On the other hand, we provided the rats with one kind of UPF instead of a variety to choose from.

This study addressed the new implication of UPF-based diets beyond the known metabolic effects. The exclusive consumption of UPF leads to adverse effects on bone growth and quality, with specific damage to the GP, which makes it particularly relevant to the young population, as the GP serves the engine of longitudinal growth. We show nutritional interventions dealing with this damage, as during growth, the accrual of bone mass and achieving an optimal peak bone mass are greatly important. Thereby, it directly impacts the risk of osteoporotic fractures in advanced age. Thus, nutritional components are fundamentally important during this crucial growth process. One may claim that with proper supplementation, the harmful costs of ultra-processed diets can be prevented; however, our results indicate that artificial supplementation is most probably not a safe alternative to a balanced diet.

## 4. Materials and Methods

### 4.1. Experimental Design

In vivo studies were conducted to establish the effect of a diet based on UPF on bone formation. All experiments were conducted on female Sprague Dawley (SD) rats after weaning (3 weeks of age). We chose young female rats before sexual maturation as our study model because female subjects are more prone to suffer from bone diseases. Thus, improving bone quality in young females is a preventative approach. The first experiment examined the effect of supplementation with micro-nutrients. A total of 24 female Sprague Dawley rats after weaning (3 weeks old) were purchased from Harlan Laboratories (Rehovot, Israel). They were housed in standard environmental conditions with a 12 h light and 12 h dark cycle and had ad libitum access to food, water, and soft drink. After 4 days of adaptation, the rats were randomly divided to three groups: Control diet, diet based on UPF with caloric soft drink (UPF + CSD), and diet based on UPF with multi-vitamin and mineral supplement and CSD (UPF + CSD + MV). During this experiment, we consistently found significant deficiencies in calcium (Ca) and copper (Cu) in the UPF + CSD group. Hence, we conducted a second experiment that involved supplementing the UPF diet with Ca or Cu. The third experiment examined whether a balanced diet (Control diet) could repair existing disturbances in growth following the consumption of an unbalanced diet and growth retardation. Exclusive consumption of UPF for 3 weeks was followed by the consumption of the Control diet before growth cessation in short and prolonged repair during the accelerated growth period (1 week and 3 weeks of Control diet following 3 weeks of UPF diet). All procedures were approved by the Hebrew University Animal Care Committee #AG-13-13952-2, AG-18-15441-2.

Throughout the experiment, body weight and food intake were measured twice a week, and the food consumption (grams and kcal) was calculated for each rat per day. After 6 weeks, the animals were anesthetized with isoflurane, blood samples were collected, and the animals were sacrificed. Their organs (kidney) and bones (femur, tibia, and vertebra) were harvested. The femur, tibia, and vertebra were manually cleaned of soft tissue and stored until testing at −20 °C (for mechanical/µCT and mechanical testing) or were fixed immediately after sacrifice (for histological studies).

Diet Preparation and Composition

The diets supplied during the experiment were as follows:

1. Control diet: 20% protein, 16% fat and 64% carbohydrate.
2. Ultra-processed diet: 20% protein, 40% fat and 40% carbohydrate.
3. Ultra-processed diet with multivitamin and mineral supplement: 20% protein, 40% fat and 40% carbohydrate; supplemented with multi vitamins and minerals (AIN-93-VX Vitamin Mix and AIN Mineral Mixture 76), while the Ca content was the guideline for supplementation and equal to the Control.
4. Ultra-processed diet with Ca supplement: 20% protein, 40% fat and 40% carbohydrate; supplemented with Ca phosphate according to the calcium content of the Control chew diet.
5. Ultra-processed diet with copper supplement: 20% protein, 40% fat and 40% carbohydrate; supplemented with copper carbonate basic according to Harlan’s copper content of the Control chew diet.

The ultra-processed diet was purchased from a popular fast-food chain (McDonald’s, Rehovot, Israel) and included a roll of bread, a hamburger, tomatoes, lettuce, ketchup (without onion and pickles), and French fries. The whole meal was homogenized, shaped, as dumplings and frozen at −20 °C. The soft drink was Coca-Cola, a typical caloric soft drink. Throughout the experiment body weight and food intake were measured twice a week, and the food consumption (grams and kcal) was calculated for each rat per day.

### 4.2. Diet Analysis

The macro and micro-nutrient content of the Control and ultra-processed diets was analyzed by the Aminolab Laboratory (Rehovot, Israel), which provides analytical services certified by the FDA and the Israeli Ministry of Health (Figure S1). This analysis was compatible with the information provided by Harlan Laboratories (Rehovot, Israel) for the Control diet, and the nutritional database of the United States Department of Agriculture (www.ndb.nal.usda.gov/ndb/ accessed on 20 November 2021) for the ultra-processed diet. Calories from each macro-nutrient were obtained by multiplying the grams of protein, carbohydrates, and fats by the factors, 4-4-9, respectively [46].

### 4.3. Blood Analysis

Blood samples were collected at the time of the sacrifice for a hematological analysis (complete blood count) in the Veterinary Teaching Hospital in Bet Dagan. The blood was centrifuged, and the serum was collected and analyzed for a complete biochemical profile. Additionally, a hormonal profile analysis was conducted in the American Medical Laboratories (Herzliya, Israel).

### 4.4. Histological Analyses of the Growth Plate and Kidneys

The effect of the different diets on the growth plates (GP) of growing rats was examined by histological techniques (to determine structure and cell types). Histological staining included hematoxylin and eosin (H&E), Safranin-O, alkaline phosphatase (ALP), and tartrate-resistant acidic phosphatase (TRAP) [47,48]. Alcian Von Kossa staining (AgNO3 2%) was performed to mineral deposits in the kidneys [49]. The widths of the whole GP, and the proliferative zone (PZ), hypertrophic zone (HZ), and lesion zone (LZ) were measured at 10 different points along the GP and averaged with measurements from 10 other plate samples in each group. The percentage of PZ and of HZ/LZ from the whole GP was calculated. Stained tissue sections were viewed under an Eclipse E400 Nikon light microscope (Nikon, Tokyo, Japan) at various magnifications using light filters. Pictures were taken with an Olympus DP71 camera controlled by Cell A software (Olympus, Tokyo, Japan) [50].

### 4.5. Skeleton Analyses

#### 4.5.1. Micro Computed Tomography (µCT) Analysis

Femora and lumbar vertebrae were scanned in a Skyscan 1174 X-ray computed microtomography scanner device (Brucker, Kontich, Belgium). Images were obtained with a 50 kV X-ray tube voltages and 800 μA current. The bones were scanned using a 0.25 mm aluminum filter at a 2500 ms exposure time and at an isotropic voxel size of 15.1 μm. For each specimen, a series of 900 projection images was obtained with a rotation step of 0.4°, averaging 2 frames, for a total of 360° rotation. The region of interest was established by a preserved starting point, which was defined as a relative distance from a reproducible landmark. For analyses of the diaphyseal cortical region, the mid-diaphysis was selected, and 200 slices corresponding to 2.764 mm were chosen. Global grayscale threshold levels for the cortical region were selected. For trabecular analysis of the secondary ossification zone, a total of 150 slices above the area of the metaphyseal GP, was selected, which corresponded to 2.073 mm, and adaptive grayscale threshold levels were used [51,52].

#### 4.5.2. Light Microscopy Images of Cross-Sections of Rat Cortical Bone

Transverse slices of a similar thickness (approximately 1.2 mm) were prepared from the mid-diaphysis of the left femora. Each slice was marked, so its axial position as well as its proximal, medial, and cranial aspects could be identified. Cross- sectional slices were prepared with a diamond-blade water-cooled saw (Isomet^®^ low speed saw, Buhler, Plymouth, MN, USA). Then, the slices were ground and polished by successively using more refined abrasive paper (from 2500 to 4000 grit) followed by the use of a 3 and 1 µm cloth with diamond paste [53]. The polished surfaces were studied using reflected-light microscopy (Olympus^®^ BX-51 microscope, Olympus, Tokyo, Japan). Images were captured using a 12.1 megapixel-resolution camera attached to the microscope (Olympus^®^ DP 71 digital camera, Olympus, Tokyo, Japan).

#### 4.5.3. Mechanical Testing

Biomechanical testing was performed using an Instron material testing machine (Model 3345) fitted with a custom-built testing chamber containing saline. All bones were tested by the three-point bending method while fully immersed in saline. Each bone was placed on two supports with rounded profiles, such that the supports were equidistant from the ends of the bone. Each bone was loaded on its lateral aspect at the mid-point between the bottom supports and at the precise mid-point along its length. Loading proceeded at a constant rate (600 μm/min) up to the fracture point, as identified by a sudden decrease in load. Force–displacement data were collected by Instron software (BlueHill) at 10 Hz. The resulting load–displacement curves were used to calculate whole bone stiffness, yield load, load to fracture, ultimate load, and area under the curve (AUC) for the calculation of the total energy to fracture (E to fracture) [47,54,55].

### 4.6. RNA Isolation, Reverse Transcription, and Real-Time PCR

An amount of 100 mg of kidney tissue was collected (3 from each group) and homogenized in TRI Reagent (Sigma, St. Louis, MO, USA). Total RNA was extracted according to the manufacturer’s protocol. RNA (1 µg) was reverse-transcribed using a high-capacity cDNA reverse transcription kit (Applied Biosystems, Foster City, CA, USA). Relative quantification real-time PCR was performed using platinum SYBR Green, 1 µL of cDNA template, and a specific primer set for each gene of interest [47].

TRPV5 (F) CTTACGGGTTGAACACCACCA, (R) TTGCAGAACCACAGAGCCTCTA

Calbindin-D28k (F) GGAAGCTGGAGCTGACAGAGAT,

(R) TGAACTCTTTCCCACACATTTTGAT

CYP27B1 (F) GAGATCACAGGCGCTGTGAAC, (R) TCCAACATCAACACTTCTTTGATCA

CYP24 (F) TGGATGAGCTGTGCGATGA, (R) TGCTTTCAAAGGACCACTTGTTC

MCP-1 (F) TATGCAGGTCTCTGTCACGC, (R) AAGTGTTGAACCAGGATTCACA

FGFR1 (F) ATACCACCGACAAGGAAATG, (R) TTCCAGGTACAGAGGTGAGG

Klotho (F) CAATGGCTTCCCTCCTTTAC, (R) AGCACAGGTTTGCGTAGTCT

18S (F) CGCGGTTCTATTTTGTTGGT, (R) AGTCGGCATCGTTTATGGTC.

### 4.7. Statistical Analysis

All data are expressed as mean ± STDEV (standard deviation). The significance of differences between groups was determined using JMP 14.0 Statistical Discovery Software (SAS Institute 2000) by one-way analysis of variance. Differences between groups at each time point were further evaluated by a Tukey–Kramer HSD test. Differences were considered significant at *p* ≤ 0.05.

## Figures and Tables

**Table 1:** Consumption of ultra-processed diet leads to alterations in bone architecture and biomechanical properties.

*The Tested Parameter*	Calcium Supplementation Experiment			Copper Supplementation Experiment		
***Trabeculacr analysis of the femur***	**Control**	**UPF + CSD**	**UPF + CSD + Ca**	**Control**	**UPF**	**UPF + Cu**
BV/TV (%)	27.37 ± 4.61 ^a^	16.61 ± 3.85 ^b^	24.86 ± 2.73 ^ab^	31.26 ± 3.33 ^a^	18.47 ± 3.82 ^b^	18.07 ± 2.54 ^b^
Tb.N (1/mm)	2.41 ± 0.25 ^a^	1.76 ± 0.40 ^b^	2.07 ± 0.19 ^ab^	2.65 ± 0.24 ^a^	2.27 ± 0.41 ^b^	2.23 ± 0.39 ^b^
Tb.Th (mm)	0.11 ± 0.008 ^ab^	0.09 ± 0.003 ^b^	0.12 ± 0.006 ^a^	0.12 ± 0.004 ^a^	0.08 ± 0.003 ^b^	0.08 ± 0.01 ^b^
Tb.Sp (mm)	0.31 ± 0.038 ^b^	0.53 ± 0.18 ^a^	0.42 ± 0.09 ^b^	0.28 ± 0.03 ^b^	0.56 ± 0.17 ^a^	0.43 ± 0.02 ^a^
***Trabeculacr analysis of the vertebra***	**Control**	**UPF + CSD**	**UPF + CSD + Ca**	**Control**	**UPF**	**UPF + Cu**
BV/TV (%)	40.37 ± 3.78 ^a^	26.50 ± 3.55 ^c^	34.05 ± 2.48 ^b^	42.9 ± 2.82 ^a^	21.06 ± 1.63 ^b^	22.62 ± 2.96 ^b^
Tb.N (1/mm)	4.73 ± 0.58 ^a^	3.53 ± 0.42 ^b^	4.03 ± 0.28 ^b^	2.87 ± 0.14 ^a^	1.97 ± 0.11 ^b^	2.00 ± 0.16 ^b^
Tb.Th (mm)	0.086 ± 0.003 ^a^	0.075 ± 0.004 ^b^	0.085 ± 0.006 ^a^	0.15 ± 0.01 ^a^	0.11 ± 0.003 ^b^	0.11 ± 0.01 ^b^
Tb.Sp (mm)	0.23 ± 0.03 ^b^	0.30 ± 0.03 ^a^	0.27 ± 0.01 ^a^	0.26 ± 0.02 ^b^	0.35 ± 0.02 ^a^	0.35 ± 0.02 ^a^
***Cortical analysis of the femur***	**Control**	**UPF + CSD**	**UPF + CSD + Ca**	**Control**	**UPF**	**UPF + Cu**
Ct.Ar/Tt.Ar (%)	46.62 ± 3.93 ^b^	31.34 ± 3.87 ^c^	51.29 ± 2.12 ^a^	48.61 ± 2.23 ^a^	33.59 ± 3.72 ^b^	36.23 ± 3.17 ^b^
Ct.Th (mm)	0.47 ± 0.03 ^a^	0.24 ± 0.02 ^b^	0.50 ± 0.02 ^a^	0.44 ± 0.02 ^a^	0.23 ± 0.02 ^c^	0.26 ± 0.02 ^b^
Ma.Ar (mm^2^)	6.07 ± 0.7 ^a^	6.67 ± 0.95 ^a^	4.91 ± 0.37 ^b^	4.63 ± 0.39 ^b^	5.34 ± 0.71 ^a^	5.12 ± 0.29 ^ab^
BMD (g/cm^3^)	1.10 ± 0.02 ^b^	0.81 ± 0.05 ^c^	1.17 ± 0.01 ^a^	1.54 ± 0.03 ^a^	1.08 ± 0.04 ^b^	1.03 ± 0.04 ^c^
Ct.Po (%)	0.06 ± 0.05 ^b^	2.3 ± 0.83 ^a^	0.02 ± 0.02 ^b^	0.13 ± 0.10 ^c^	4.61 ± 1.29 ^a^	3.30 ± 1.15 ^b^
Po.N (n)	3.83 ± 2.14 ^b^	21 ± 7.42 ^a^	1.83 ± 0.98 ^b^	1.6 ± 1.07 ^b^	34.45 ± 17.10 ^a^	28.00 ± 14.35 ^a^
Po.V (mm^3^)	0.01 ± 0.01 ^b^	0.2 ± 0.1 ^a^	0.003 ± 0.003 ^b^	0.02 ± 0.01 ^b^	0.33 ± 0.10 ^a^	0.26 ± 0.09 ^a^
***Three point bending test***	**Control**	**UPF + CSD**	**UPF + CSD + Ca**	**Control**	**UPF**	**UPF + Cu**
Stiffness (N/mm)	239.56 ± 45.30 ^a^	36.18 ± 14.38 ^b^	282.44 ± 39.52 ^a^	273.77 ± 23.53 ^a^	51.59 ± 13.70 ^b^	40.64 ± 7.88 ^b^
yield (N)	49.49 ± 3.07 ^a^	17.47 ± 2.81 ^b^	50.94 ± 6.88 ^a^	42.02 ± 4.38 ^a^	14.02 ± 2.50 ^b^	14.77 ± 1.78 ^b^
fracture load (N)	72.00 ± 10.14 ^a^	12.86 ± 7.28 ^b^	71.56 ± 12.52 ^a^	74.95 ± 10.08 ^a^	18.11 ± 6.65 ^b^	16.78 ± 6.66 ^b^
Max load (N)	79.31 ± 8.40 ^a^	22.03 ± 3.45 ^b^	82.18 ± 8.04 ^a^	87.04 ± 8.16 ^a^	25.28 ± 3.76 ^b^	23.35 ± 3.33 ^b^
Energy to fracture (Nxmm)	54.29 ± 7.39 ^a^	36.14 ± 11.58 ^b^	50.23 ± 7.92 ^a^	66.57 ± 14.35 ^a^	40.86 ± 7.82 ^b^	41.35 ± 6.50 ^b^

Femora and vertebrae of 9-week-old rats were subjected to µCT scan. Trabecular bone parameters: bone volume fraction (BV/TV), trabecular number (Tb.N), trabecular separation (Tb.Sp), and trabecular thickness (Tb.Th). Cortical bone parameters: cortical area fraction (Ct.Ar/Tt.Ar), average cortical thickness (Ct.Th), medullary area (Ma.Ar), bone mineral density (BMD), percentage of object volume (Obj.V/TV), cortical porosity (Ct.Po), pore number (Po.N), and total pore volume (Po.V). Three-point bending was used to measure the biomechanical properties of femora, which was derived from load–displacement curves. Values are expressed as mean ± SD, n = 8. Different letters denote significant difference at *p* < 0.05 between groups.

## Data Availability

Not applicable.

## Supplementary Materials

The following supporting information can be downloaded at: https://www.mdpi.com/article/10.3390/ijms23020841/s1.
