# Supplementary material for: Nutritional Approaches as a Treatment for Impaired Bone Growth and Quality Following the Consumption of Ultra-Processed Food
Source: Int J Mol Sci. 2022 Jan 13;23(2):841. doi: 10.3390/ijms23020841 (PMC8776230; doi:10.3390/ijms23020841)
Supplement: Supplementary file 1 [file ijms-23-00841-s001.zip › ijms-1542927-supplementary.pdf]

Supplementary Figures and Tables

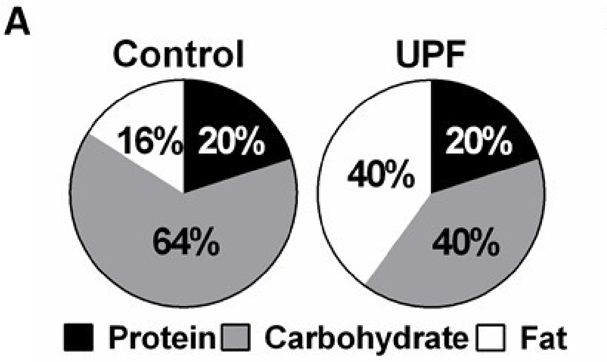

B

Micro-nutrients (per 100 gr)	Control	UPF
**Calcium** (mg)	1000	31.9
**Phosphorus** (mg)	700	137.7
**Zinc** (mg)	7	1.7
**Iron** (mg)	20	1.3
**Magnesium** (mg)	200	26.2
**Copper** (mg)	1.5	0.1

Supplementary Figure S1. Schematic illustration of diet composition. (**A**) Caloric distribution of macronutrients in the Control diet and in the UPF diet. (**B**) Micronutrient composition of calcium, phosphorus, zinc, iron, magnesium, and copper in the Control diet compared with the UPF diet.

Supplementary Table S1. Serum analysis.

A

	Blood parameters	Control	UPF+CSD	UPF+CSD+MV
Metabolic parameters	Glucose (mg/dl)	152.81 ± 25.42	157.66 ± 41.27	124.44 ± 19.55
	Triglycerides (mg/dl)	79.98 ± 8.11	88.97 ± 37.12	62.50 ± 17.86
	Cholesterol (mg/dl)	130.89 ± 18.19^{a}	114.12 ± 13.1^{ab}	93.36 ± 18.75^{b}
	Urea (mg/dl)	37.11 ± 5.66^{a}	25.53 ± 3.20^{b}	27.56 ± 4.38^{b}
	Creatinine (mg/dl)	0.40 ± 0.08^{a}	0.36 ± 0.07^{ab}	0.29 ± 0.06^{b}
	Creatine Phosphokinase (IU/L)	187.63 ± 34.08^{b}	1631.63 ± 1252.68^{a}	584.43 ± 251.71^{b}
	α-Amylase (IU/L)	1203.71 ± 247.04^{b}	1781.84 ± 487.78^{a}	1064.85 ± 168.67^{b}
	Chloride (IU/L)	112.8 ± 15.26	99.89 ± 9.1	101.24 ± 7.02
	Sodium (IU/L)	150.51 ± 19.38	132.80 ± 13.48	137.48 ± 9.66
	Potassium (IU/L)	9.04 ± 1.04^{a}	6.94 ± 1.28^{b}	7.06 ± 0.57^{b}
Bone parameters	Phosphate (mg/dl)	9.21 ± 1.57^{b}	12.71 ± 1.64^{a}	7.93 ± 0.76^{b}
	Calcium (mg/dl)	11.55 ± 1.42^{a}	5.27 ± 0.76^{b}	10.98 ± 1.05^{a}
	Alkaline Phosphatase (IU/L)	84.78 ± 7.91^{b}	209.21 ± 50.90^{a}	73.75 ± 15.4^{b}
Liver function parameters	Albumin (g/dl)	4.29 ± 0.53	4.18 ± 0.39	3.90 ± 0.33
	Total Protein (g/dl)	5.76 ± 0.31	5.79 ± 0.44	5.56 ± 0.45
	Alanine Transaminase (IU/L)	38.5 ± 4.83^{b}	63.05 ± 6.68^{a}	32.73 ± 5.06^{b}
	Aspartate Transaminase (IU/L)	101.47 ± 11.00^{b}	282.5 ± 151.35^{a}	154.29 ± 31.31^{b}

B

	Control	UPF+CSD	UPF+CSD+MV
ACTH (pg/ml)	891.93±656.84	454.64±216.86	750.98±350.88
Leptin (pg/ml)	663.91±70.67	473.48±191.85	742.94±282.13
PTH (pg/ml)	581.20±225.25^{b}	2584.02^{a}	361.87±121.48^{b}
OPG (pg/ml)	587.71±191.51	474.32±110.78	443.25±36.56
DKK1 (pg/ml)	1822.54±464.89^{a}	512.66±246.19^{b}	1414.35±377.11^{ab}
Sclerostin (pg/ml)	366.74±65.71^{a}	108.77±72.87^{b}	460.40±25.05^{a}
FGF23 (pg/ml)	1812.51±351.68^{a}	480.30±34.50^{b}	1379.74±198.36^{a}

(**A**) Serum analysis of rats and (**B**) Hormonal profile of rats from the ultra- processed diet & supplementation experiment. Values are expressed as mean ± SD. Different letters denote significant difference at $P < 0.05$ between groups.

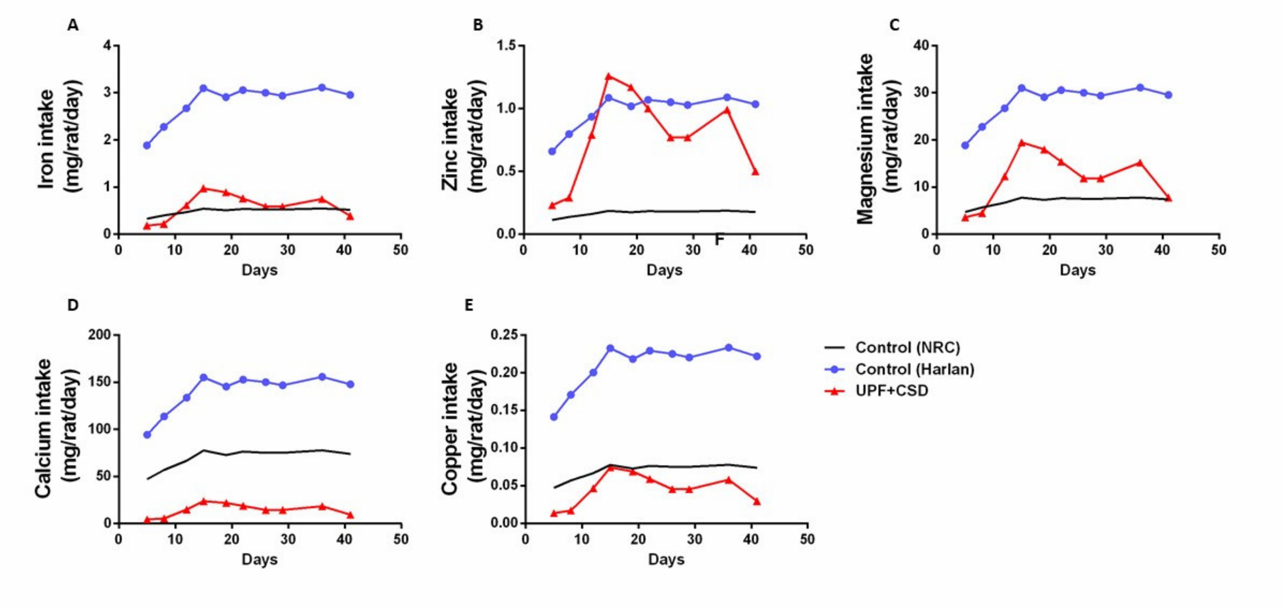

Supplementary Figure S2. Increased intake the ultra-processed food (UPF) leads to a partial compensation of the deficient minerals. Control and UPF+CSD groups were compared based on their micro-nutrient consumption. The black line represents the recommendation of mineral intake of growing rats. (A) Daily iron intake (mg/rat per day). (B) Daily zinc intake (mg/rat per day). (C) Daily magnesium intake (mg/rat per day). (D) Daily calcium intake (mg/rat per day). (E) Daily copper intake (mg/rat per day).

Supplementary Table S2. Serum analysis.

A

	Blood parameters	Control	UPF+CSD	UPF+CSD+Ca
Metabolic parameters	Glucose (mg/dl)	152.81 ± 25.42	157.66 ± 41.27	171.58 ± 30.27
	Triglycerides (mg/dl)	79.98 ± 8.11	88.97 ± 37.12	60.44 ± 33.89
	Cholesterol (mg/dl)	130.89 ± 18.19^{a}	114.12 ± 13.1^{ab}	103.73 ± 16.71^{b}
	Urea (mg/dl)	37.11 ± 5.66^{a}	25.53 ± 3.20^{b}	32.33 ± 4.15^{a}
	Creatinine (mg/dl)	0.40 ± 0.08	0.36 ± 0.07	0.38 ± 0.07
	Creatine Phosphokinase (IU/L)	187.63 ± 34.08^{b}	1631.63 ± 1252.68^{a}	661.14± 630.02^{ab}
	α-Amylase (IU/L)	1203.71 ± 247.04^{b}	1781.84 ± 487.78^{a}	1640.34 ± 151.98^{a}
	Chloride (IU/L)	112.8 ± 15.26	99.89 ± 9.1	101.68 ± 2.79
	Sodium (IU/L)	150.51 ± 19.38	132.80 ± 13.48	139.08 ± 3.27
	Potassium (IU/L)	9.46 ± 1.52^{a}	6.94 ± 1.28^{b}	6.71 ± 1.04^{b}
Bone parameters	Phosphate (mg/dl)	9.21 ± 1.57^{b}	12.71 ± 1.64^{a}	9.04 ± 0.69^{b}
	Calcium (mg/dl)	11.55 ± 1.42^{a}	5.27 ± 0.76^{b}	11.50 ± 0.54^{a}
	Alkaline Phosphatase (IU/L)	84.78 ± 7.91^{b}	209.21 ± 50.90^{a}	99.21 ± 30.19^{b}
Liver function parameters	Albumin (g/dl)	4.75 ± 0.23	4.18 ± 0.39	4.11 ± 0.26
	Total Protein (g/dl)	5.76 ± 0.31	5.79 ± 0.44	5.70 ± 0.31
	Alanine Transaminase (IU/L)	38.5 ± 4.83^{b}	63.05 ± 6.68^{a}	23.91 ± 3.71^{c}
	Aspartate Transaminase (IU/L)	101.47 ± 11.00^{b}	282.5 ± 151.35^{a}	131.16 ± 71.63^{b}

B

	Control	UPF+CSD	UPF+CSD+Ca
ACTH (pg/ml)	891.93±656.84	454.64±216.86	2081.59±1162.22
Leptin (pg/ml)	663.91±70.67	473.48±191.85	1223.85±460.02
PTH (pg/ml)	581.20±225.25^{b}	2584.02^{a}	624.97±331.93^{b}
OPG (pg/ml)	587.71±191.51	474.32±110.78	470.25±61.29
DKK1 (pg/ml)	1822.54±464.89^{a}	512.66±246.19^{b}	1429.08±171.72^{a}
Sclerostin (pg/ml)	366.74±65.71^{ab}	108.77±72.87^{b}	589.94±173.7^{a}
FGF23 (pg/ml)	1812.51±351.68^{a}	480.30±34.50^{b}	1540.86±374.98^{a}

C

	Control6W	UPF6W	UPF+Cu
Glucose (mg/dl)	190.14 ± 18.95	183.46 ± 22.28	186.21 ± 17.27
Triglycerides (mg/dl)	80.1 ± 16.49^{a}	21.51 ± 6.56^{b}	34.23 ± 10.15^{b}
Phosphorus (mg/dl)	7.08 ± 1.05^{c}	8.94 ± 0.7^{b}	10.27 ±1.06^{a}
Calcium (mg/dl)	11.02 ±0.96^{a}	6.34 ± 0.78^{b}	5.32 ± 0.73^{b}
Alkaline Phosphatase (U/L)	168 ± 23.53^{b}	374.5 ± 50.83^{a}	394.88 ± 43.15^{a}
Albumin (g/dl)	4.96 ± 0.5	5.25 ± 0.04	4.92 ± 0.28
Cholesterol (mg/dl)	120.59 ± 12.96^{a}	97.23 ± 8.41^{b}	94.35 ± 10.03^{b}
Alanine Transaminase (IU/L)	53.21 ± 4.49^{b}	61.3 ± 9.14^{b}	75.94 ± 12.1^{a}
Aspartate Transaminase (IU/L)	145.34 ± 48.49^{b}	117.65 ± 29.62^{b}	233.34 ± 60.57^{a}

(**A**) Serum and hormonal analysis of rats from the ultra- processed diet & Ca supplementation experiment. (**C**) Serum analysis of rats from the ultra- processed diet & Cu supplementation experiment. Values are expressed as mean ± SD. Different letters denote significant difference at *P* < 0.05 between groups.

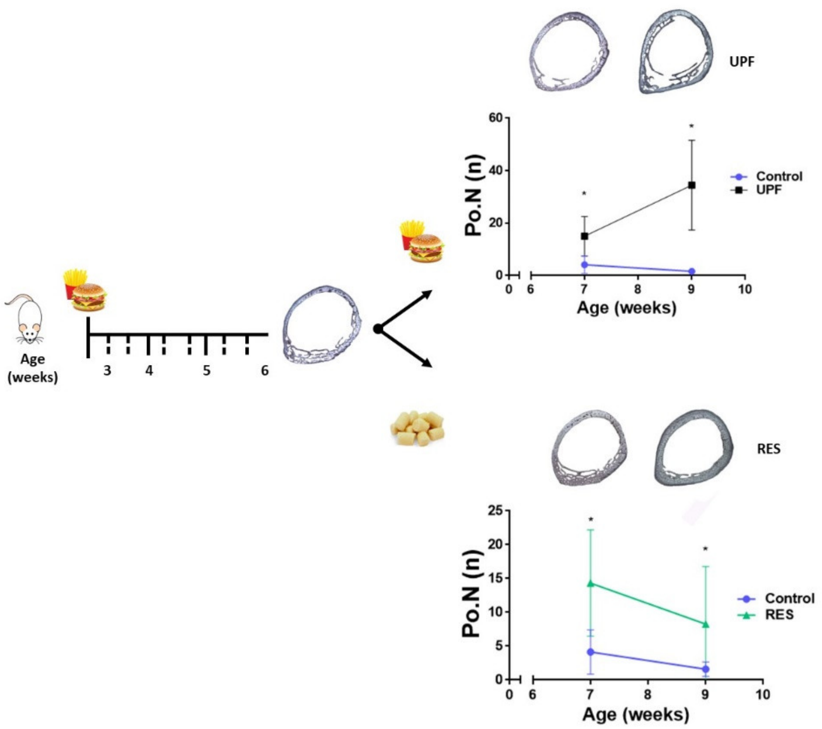

Supplementary Figure S3. Porosity levels of the cortical bone following 3 weeks of UPF intake and the change in the number of pores following continuation of UPF intake or shifting to a Control diet. Po.N values were calculated following uCT analysis. Light microscopy images of cross-sections of rat cortical bone representing cortical bone porosity. Values are expressed as mean ± SD, n = 8. *$p < 0.05$ compared to Control.

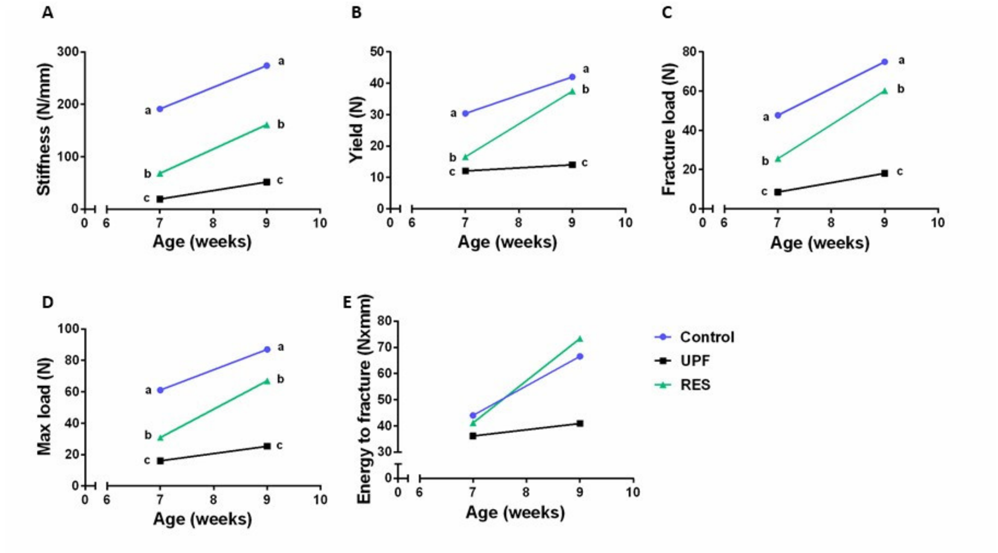

Supplementary Figure S4. Biomechanical properties of the rescue experiment. Three-point bending was used to measure the biomechanical properties of femora, derived from load-displacement curves. Values are expressed as mean ± SD, n = 8. Different letters denote significant difference at $P < 0.05$ between groups.

Supplementary Table S3. Serum analysis.

A

	Control4W	UPF4W	RES1W
Glucose (mg/dl)	208.65 ± 25.423	190.18 ± 11.57	205.34 ± 13.56
Triglycerides (mg/dl)	72.28 ± 14.24[a]	26.62 ± 3.99[b]	83.86 ± 16.44[a]
Phosphorus (mg/dl)	7.8 ± 1.28[b]	10.53 ± 0.64[a]	9.14 ± 0.7[ab]
Calcium (mg/dl)	11.25 ±0.42[a]	6.51 ± 0.36[b]	11.17 ± 0.3[a]
Alkaline Phosphatase (U/L)	198.2 ± 22.97[b]	426 ± 77.37[a]	242.4 ± 42.39[b]
Albumin (g/dl)	4.86 ± 0.1[b]	5.2 ± 0.09[a]	4.77 ± 0.11[b]
Cholesterol (mg/dl)	137.9 ± 13.57[a]	99.44 ± 9.41[b]	127.6 ± 10.1[a]
Alanine Transaminase (IU/L)	50.88 ± 12.66[b]	83.28 ± 22.01[a]	49.82 ± 8.16[b]
Aspartate Transaminase (IU/L)	168.54 ± 109.14	195.02 ± 100.45	136.42 ± 46.06

B

	Control4W	UPF4W	RES1W
OPG (pg/ml)	826.5±139.71	736.17±248.64	804.8±243.75
PTH (pg/ml)	412±174.88[b]	1823.67±364.09[a]	744.4±291.2[b]
FGF23 (pg/ml)	473.6±156.17[a]	102.17±51.16[b]	375±102.88[a]

C

	Control6W	UPF6W	RES3W
Glucose (mg/dl)	190.14 ± 18.95	183.46 ± 22.28	210.68 ± 22.28
Triglycerides (mg/dl)	80.1 ± 16.49[a]	21.51 ± 6.56[b]	73.18 ± 28.08[a]
Phosphorus (mg/dl)	7.08 ± 1.05[b]	8.94 ± 0.7[a]	7.51 ±0.62[b]
Calcium (mg/dl)	11.02 ±0.96[a]	6.34 ± 0.78[b]	10.83 ± 0.46[a]
Alkaline Phosphatase (U/L)	168 ± 23.53[b]	374.5 ± 50.83[a]	189.75 ± 15.0[b]
Albumin (g/dl)	4.96 ± 0.5	5.25 ± 0.04	5.01 ± 0.15
Cholesterol (mg/dl)	120.59 ± 12.96[a]	97.23 ± 8.41[b]	119.08 ± 4.52[a]
Alanine Transaminase (IU/L)	53.21 ± 4.49	61.3 ± 9.14	52.25 ± 5.82
Aspartate Transaminase (IU/L)	145.34 ± 48.49	117.65 ± 29.62	121.8 ± 50.27

D

	Control6W	UPF6W	RES3W
OPG (pg/ml)	637.67±136.62	526±86.7	589.2±164.5
PTH (pg/ml)	581.20±225.25[b]	2584.02[a]	361.87±121.48[b]
FGF23 (pg/ml)	1812.51±351.68[a]	480.30±34.50[b]	1379.74±198.36[a]

Serum analysis of rats and from the ultra-processed diet & rescue experiment. (**A**,**B**) short rescue. (**C**,**D**) prolonged rescue. Values are expressed as mean ± SD. Different letters denote significant difference at $P < 0.05$ between groups.
